# Delivery timing determines senomorphic efficacy: delayed local ruxolitinib restores macrophage polarization and rescues age-impaired bone repair

**DOI:** 10.21203/rs.3.rs-9624236/v1

**Published:** 2026-05-22

**Authors:** Yamin Liu, Erin M. O’Brien, Christy Lottinger, Govindaraj Perumal, Kara L. Spiller, Liisa Kuhn

**Affiliations:** University of Connecticut Health Center; Drexel University; University of Connecticut Health Center; University of Connecticut Health Center; Drexel University; University of Connecticut Health Center

## Abstract

Impaired bone repair in aged individuals is driven by dysregulated macrophage polarization and chronic inflammaging. Early pro-inflammatory macrophage activity is indispensable for bone repair, yet its pathological persistence suppresses osteogenesis and promotes bone resorption. Here we show that localized, temporally delayed delivery of the JAK1/JAK2 inhibitor ruxolitinib (Rux) from a transitory barrier layer (TBL) bone graft coating restores macrophage phenotype switching and accelerates calvarial bone defect closure in aged female mice. Immediate Rux exposure prematurely suppresses the early pro-inflammatory gene expression profiling in aged bone marrow-derived macrophages, whereas TBL-mediated delayed release preserves this phase before driving a robust pro-reparative gene expression signature. Rux-TBL-coated grafts produced significantly greater bone formation than immediately dosed Rux grafts or no Rux grafts at nine weeks. These findings establish delivery timing as a critical determinant of senomorphic efficacy, supporting local delayed delivery over systemic administration following acute bone injury.

## Introduction

Bone injuries heal markedly more slowly in elderly individuals^1–3^, a clinical problem of substantial and growing importance given global population aging. Conventional mechanical fixation strategies frequently fail in osteoporotic bone owing to reduced mineral density, fatty marrow infiltration, and attenuated biological repair capacity^4,5^. The sequelae of fractures in older women—including prolonged immobility, loss of independence, and excess mortality^6^—underscore the urgent need for biological strategies that actively accelerate aged bone repair.

Macrophages are central orchestrators of the bone repair cascade^7–9^. Following bone injury, a temporally regulated program of macrophage polarization unfolds: an initial pro-inflammatory (M1-like) phase facilitates the recruitment of osteoprogenitor cells and osteoclast precursors, before a transition to reparative (M2-like) phenotypes that promote matrix deposition and new bone formation^10,11,12^. In aged individuals, this polarization sequence is disrupted by inflammaging—a chronic, low-grade inflammatory state associated with the accumulation of senescent cells exhibiting a senescence-associated secretory phenotype (SASP)^5,7,13–16^. Incomplete M1-to-M2 switching in aged macrophages favors osteoclastic activity over osteoblastic differentiation, thereby reducing net bone deposition and impairing bone injury repair^16–18^.

These age-associated immune deficiencies have motivated interest in immunomodulatory therapeutic strategies. Dasatinib and quercetin treatment or Ruxolitinib (Rux), a JAK1/JAK2 inhibitor approved for the treatment of myelofibrosis, reduces SASP-associated inflammation and has been shown to improve bone mineral density and reduce frailty in aged rodent models^19,20^. Clinical trials of patients taking senolytics and/or senomorphics systemically (orally or by injection) on a continuous basis for osteoporosis management show a moderate benefit^21^. However, emerging side effects of the dasatinib and quercetin treatment on other tissues beyond bone such as brain indicates some caution must be used with systemic dosing^22^. Furthermore, the systemic use of Rux is associated with immunosuppression^23^—a particularly concerning adverse effect in older patients with diminished infectious immunity. We reasoned that locally confined delivery of the Rux directly within a bone defect could harness its senomorphic benefits while minimizing systemic exposure.

A further constraint on therapeutic design is the requirement to preserve, rather than suppress, the early pro-inflammatory phase of repair. Non-steroidal anti-inflammatory drugs (NSAIDs) and glucocorticoids are known to impair fracture healing when administered acutely after injury, and clinical guidelines recommend delaying anti-inflammatory therapy by several days following acute bone trauma^24,25^. Whether analogous timing constraints apply to senomorphic agents has not previously been considered.

To address both spatial and temporal requirements simultaneously, we developed a bone graft coating based on a transitory barrier layer (TBL) technology that achieves a delay of approximately three days before drug release^26,27^. The TBL consists of a desiccation-cracked nanocrystalline carbonated apatite deposited over an adsorbed insoluble drug layer; cellular protrusions penetrate the micro-cracks to access the sequestered drug only after direct cell–surface contact is established^28^. We hypothesized that TBL-mediated delayed Rux delivery would preserve early macrophage-driven osteoprogenitor recruitment while subsequently suppressing the pathological inflammatory state that overstimulates osteoclasts and impedes aged bone repair. Using in vitro gene expression profiling of aged murine BMDMs and a critical-sized calvarial defect model in 25-month-old mice, we demonstrate that delivery timing is a decisive determinant of Rux efficacy, and that delayed local delivery—but not immediate delivery—promotes robust new bone formation in an aged host.

## Results

### TBL coating achieves delayed release of ruxolitinib and preserves early pro-inflammatory macrophage activity

**Figure 1:**
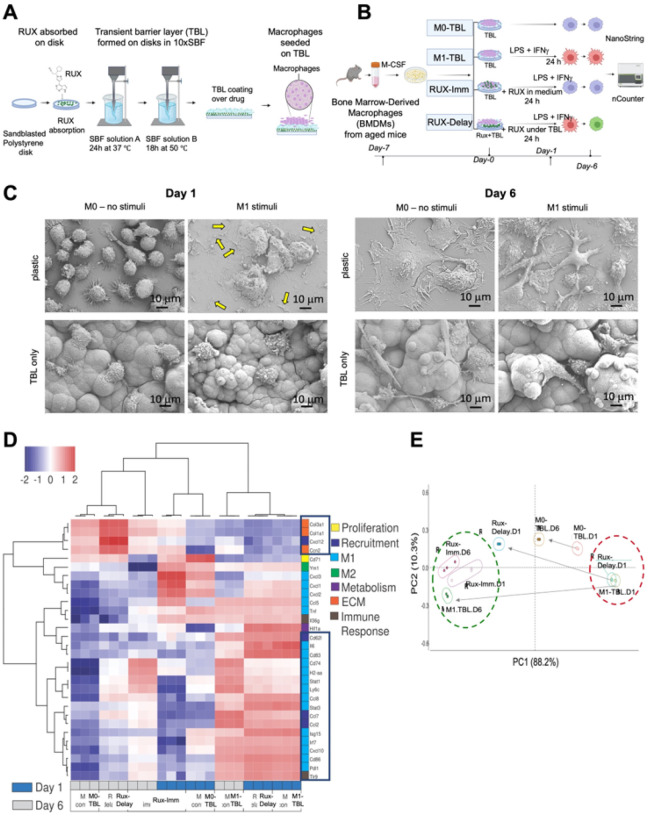
Aged macrophage in vitro response to immediate or delayed access to Ruxolitinib. **A** Schematic illustrating the TBL coating process. **B** Schematic illustrating the Nanostring experiment design and data analysis. BMDMs were extracted from aged female C57BL/6 mice and differentiated into M0 macrophages with M-CSF, then seeded on TBL coated disks. M1 stimuli (lipopolysaccharide and interferon-gamma) was applied to three groups: No Rux, Rux-Imm group (ruxolitinib in media); Rux-Delay group, (ruxolitinib under TBL). RNA was collected on day1 and 6 for Nanostring gene expression analysis. **C** SEM images of aged female murine BMDMs on tissue culture plastic (first row) and TBL coated disks (second row) with no stimuli M0 or M1 stimuli at days 1 and 6. **D** Heat map and dendrogram of the z-scored top 30 differentially expressed genes compared to M0 day 1 control. Nanostring data was normalized to total gene counts and internal controls, and Benjamini-Yekutieli p-value adjustment was used to determine and rank DEGs. **E** Principal component analysis of gene count data of M0, M1, Rux-Imm, Rux-Delay BMDMs at D1 and D6.

To investigate the consequences of delivery timing on macrophage behavior, we compared two experimental conditions: ruxolitinib added directly to culture medium (Rux-Imm), modeling the kinetics of systemic administration, and ruxolitinib sequestered beneath a transitory barrier layer (Rux-Delay), which enforces an approximately three-day lag before cellular drug access (Fig. 1A). The TBL is formed by sequential immersion of drug-coated substrates in concentrated simulated body fluid solutions, producing a nanocrystalline carbonated apatite overlayer with characteristic desiccation-induced micro-cracks^29^; prior work established that coating dissolution combined with cellular protrusions that penetrate these cracks allows access the underlying insoluble drug^26,27^.

Bone marrow-derived macrophages (BMDMs) isolated from aged (24-month-old) female C57BL/6 mice were differentiated to the M0 state and seeded onto TBL-coated substrates before stimulation with an M1 stimuli: LPS and IFN-γ to induce a pro-inflammatory phenotype (Fig. 1B). Scanning electron microscopy at day 1 revealed that cytoskeletal spreading was more restricted on TBL substrates than on non-treated tissue culture plastic (TCP), and that a subset of M1-stimulated BMDMs—particularly those on TCP—underwent cell death evidenced by remnants of cell membrane encircling where cells had been (Fig. 1C, arrows). Following withdrawal of the M1 stimuli, viable BMDMs on both substrates adopted a flattened elongated morphology by day 6, consistent with reduced inflammatory activation, though morphology alone does not resolve macrophage polarization state.

Transcriptional profiling by a custom 255-gene NanoString panel^30^ revealed a striking divergence between the two delivery conditions evaluated at two time points. At day 1, Rux-Imm broadly suppressed the pro-inflammatory macrophage-associated gene expression program, with significant downregulation of canonical pro-inflammatory transcripts across the top 30 differentially expressed genes identified by unsupervised hierarchical clustering (Fig. 1D). The statistical differences between groups of the top 30 genes in the heat mapped data is provided in Supplementary Data Table 1 (Day 1) and Table 2 (Day 2). Notably, however, Rux-Imm also elevated *Cxcl1, Cxcl2*, and *Cxcl3*—chemokines critical for osteoprogenitor recruitment. In contrast, the day 1 gene profile of Rux-Delay BMDMs was indistinguishable from that of untreated M1-stimulated controls on TBL (Fig. 1D), confirming that the TBL coating effectively occludes drug access during the early pro-inflammatory window.

The temporal dynamics were inverted by day 6. The initial anti-inflammatory effect of Rux-Imm had largely dissipated: *Cd74, H2-aa, Stat1*, and *Ly6c* returned to M1-TBL control levels, and the reparative marker Ym1 was reduced. Residual suppression of *Il6, Cd83, Stat 3* and *Isg15* was observed in Rux-Imm relative to the M1-TBL control. In contrast, Rux-Delay had driven a sustained anti-inflammatory shift at day 6, with reduced expression of many of the M1-associated genes and concomitant upregulation of extracellular matrix genes needed for bone repair—*Col3a1, Col1a1*, and *Ccn2*—relative to Rux-Imm. Principal component analysis corroborated these findings, with the large day 1 separation between Rux-Imm and M1-TBL confirming premature immunosuppression, and the convergence of day 1 Rux-Delay with the M1-TBL confirming effective drug occlusion by the TBL (Fig. 1E). The greater divergence of Rux-Delay from the M1 control at day 6, compared with Rux-Imm, further quantified the superior and delayed anti-inflammatory efficacy of TBL-mediated delivery.

### Delayed ruxolitinib delivery selectively amplifies reparative macrophage genes

**Figure 2:**
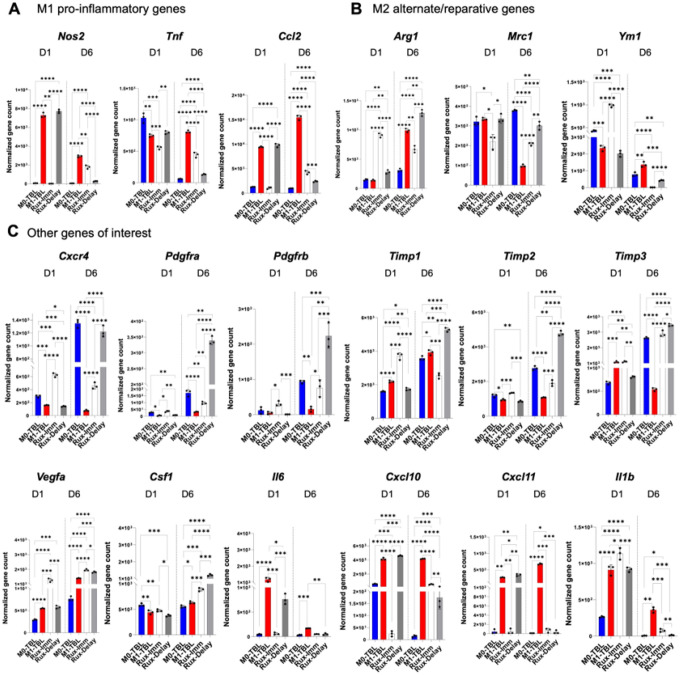
**A**. Gene counts of pro-inflammatory M1 macrophage genes on days 1 and 6. **B**Gene counts of reparative M2 macrophage genes on days 1 and 6. **C** Gene counts of other selected relevant genes on days 1 and 6. Gene differential expression analysis was performed using multiple t-tests with FDR-adjusted p-values in GraphPad Prism. Data is represented as mean ±SD.* p<0.05; ** 0.00 1£ p<0.01; *** 0.0001£ p<0.001; **** p<0.0001.

To resolve the timing-dependent gene profile effects of Rux at the level of individual macrophage polarization markers, gene counts specific for canonical markers expressed after M1 and M2 stimuli and genes associated with macrophage aging were examined (Fig. 2). Expression of the pro-inflammatory effectors *Nos2*, *Tnf*, and *Ccl2* was markedly suppressed by Rux-Imm at day 1 but was unaffected by Rux-Delay at this time point, reinforcing the TBL barrier function during the critical early phase of repair (Fig. 2A). By day 6, Rux-Delay drove greater downregulation of these pro-inflammatory transcripts than Rux-Imm, indicating a more complete and durable resolution of inflammation following delayed drug release.

The reparative macrophage markers *Arg1*, *Mrc1*, and *Ym1* showed a complementary pattern (Fig. 2B): at day 1, expression was higher in Rux-Imm than Rux-Delay, consistent with premature induction of a reparative macrophage-like state before pro-inflammatory signaling had been allowed to run its course. By day 6, this relationship reversed, with Rux-Delay producing greater upregulation of these pro-reparative transcripts. Analysis of other genes associated with inflammaging cytokines and chemokines further highlighted the effect of delivery timing. *Cxcr4*, a chemokine receptor implicated in aging of bone marrow cells^31,32^ was most highly expressed in Rux-Imm macrophages at day 1 but was normalized to M0-control levels in the Rux-Delay group by day 6 (Fig. 2C) similarly to *Il-6, Cxcl10, Cxcl11*. *Pdgfra, Pdgfrb, Timp1, Timp2, Timp3, Vegf, Csf1* that can enhance bone healing were all elevated maximally by Rux-Delay (Fig. 2C). Analysis of macrophage genes that support osteoblast activity indicate that Rux delivered by either modality significantly upregulate osteogenic genes *Bmp2, Tgfb1* and *IGF-1* relative to M0-TBL expression (Supplementary data, Table 3 (Day 1), Table 4 (Day 6)) further supporting use of Rux in the context of bone repair.

Together, these data demonstrate that the Rux-Delay TBL coating selectively preserves the early pro-inflammatory program while enabling a more robust delayed transition to a reparative macrophage state.

### Delayed ruxolitinib delivery from TBL-coated scaffolds accelerates calvarial bone repair in aged mice

**Figure 3:**
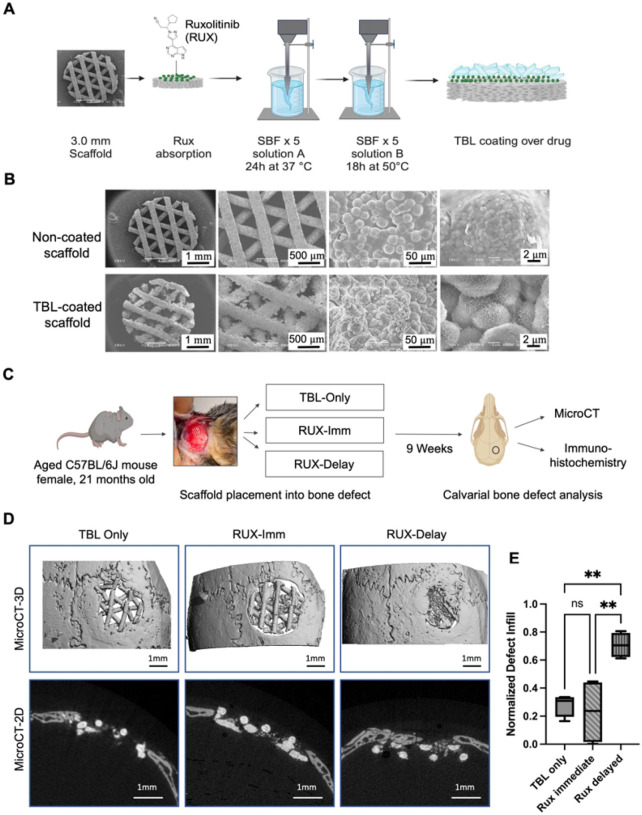
Rux-Delay prompts mouse calvarial bone defect healing. **A** Schematic of the TBL-coating process for 3D scaffolds. **B** SEM images of an uncoated and TBL-coated scaffold. **C** Experimental design of mouse calvarial bone defect experiment. **D** Representative images of mouse calvarial bone defects harvested after 9 weeks. 3-dimensional micro-CT reconstructions (middle row) and 2-dimensional micro-CT cross-sections for TBL only, Rux-Imm, Rux-Delay. **E** Quantify the new bone area in TBL only, Rux-Imm, Rux-Delay treated calvarial bone defect mice. Mean ± SD. **p < 0.01.

To evaluate the translational consequence of Rux delivery timing on bone repair, we employed a critical-sized (3.5 mm) calvarial bone defect model in 25-month-old female mice—an age broadly comparable to that of humans over 80 years. The base scaffold comprised a three-dimensional printed microporous scaffold made of three layers of filaments composed of hydroxyapatite microspheres bonded with poly(lactide-*co*-glycolide) (Hyperelastic Bone^™^, Dimension Bio), chosen for its biocompatibility, structural flexibility, and open filament architecture, which permitted uniform TBL coating throughout the scaffold interior (Fig. 3A,B). Three experimental groups were evaluated: scaffold with TBL only (no drug), scaffold with Rux applied directly at the time of implantation (Rux-Imm), and scaffold with Rux sequestered beneath the TBL coating (Rux-Delay).

Given the well-documented slowing of bone repair in aged rodents, defects were assessed at nine weeks post-implantation rather than four. Micro-computed tomography (microCT) reconstructions revealed markedly greater defect closure in the Rux-Delay group relative to both TBL-only and Rux-Imm controls (Fig. 3C,D). Quantification of new mineralized bone area confirmed that Rux-Delay produced significantly greater bone formation than either control condition (p < 0.01; Fig. 3E), whereas Rux-Imm was indistinguishable from the TBL-only scaffold, consistent with the hypothesis that immediate JAK/STAT inhibition impairs the early pro-inflammatory phase required to initiate effective repair.

### Histological analysis reveals balanced osteoblast and osteoclast activity specifically in Rux-Delay grafts

**Figure 4:**
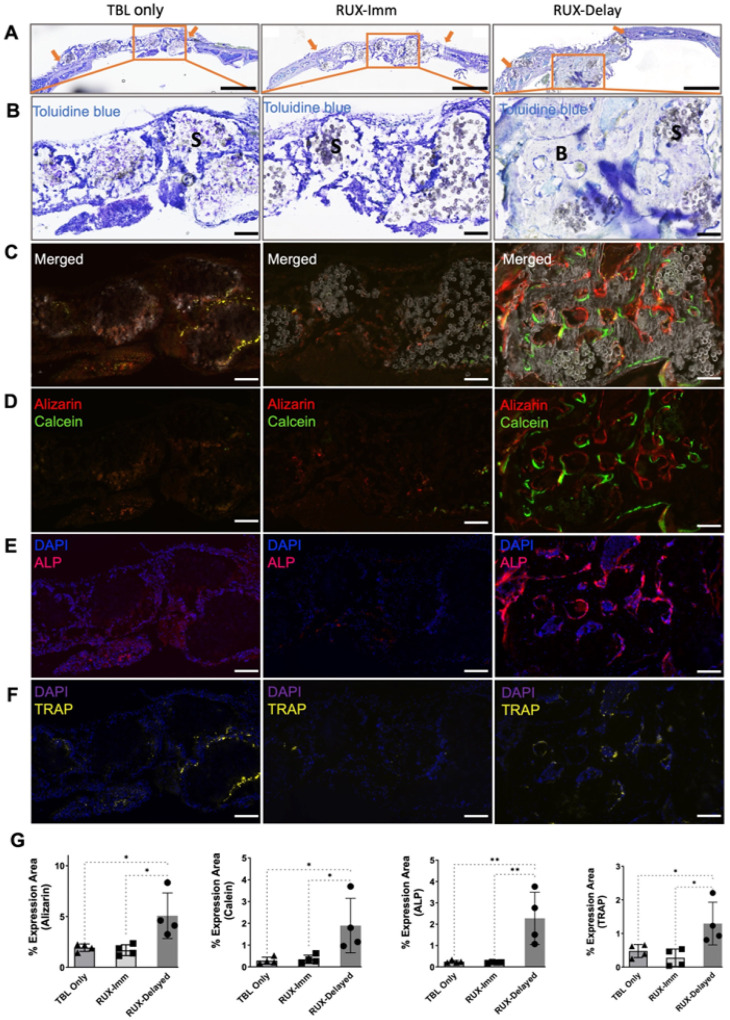
Histology images of the mouse calvarial bone defects at 9 weeks. **A** Toluidine blue stained sections for scaffold only (TBL only), TBL with immediate Rux (Rux-Imm), and TBL with delayed Rux (Rux-Delay). Scale bar = 1000 μm. **B** Higher magnification images of the yellow boxed area in the top row with new bone formation marked with “B”, scaffold marked with “S”. Scale bars = 150 μm. **C**Merged images of alizarin, calcein, ALP and TRAP staining. **D** Images of alizarin and calcein labeled calvarial bone defect sections. **E** Alkaline phosphatase (ALP) stained calvarial bone defect sections. **F** Tartrate-resistant acid phosphatase (TRAP) stained sections. **G** Quantified alizarin, calcein, ALP and TRAP staining area. Mean ± SD. *p < 0.05; ** p<0.01.

Histological examination of calvarial sections at nine weeks corroborated the microCT findings (Fig. 4). Toluidine blue staining demonstrated new woven bone (labelled ‘B’) bridging the defect in the Rux-Delay group, with minimal new bone visible in TBL-only or Rux-Imm sections (Fig. 4A,B). To distinguish between active new bone formation and passive calcification of the calcium phosphate containing scaffold material we combined fluorochrome bone labelling (alizarin complexone and calcein, administered at one and seven days before euthanasia, respectively) with enzymatic staining for alkaline phosphatase (ALP, a marker of active osteoblast function) and tartrate-resistant acid phosphatase (TRAP, a marker of osteoclast activity).

The Rux-Delay group displayed the greatest areas of alizarin, calcein, and ALP-positive staining, indicating that active osteogenesis was ongoing at the nine-week time point (Fig. 4C–E, I). Scaffold beads and lattice structure remained morphologically stable across all groups with minimal evidence of dissolution and the lack of mineral staining confirmed that mineral incorporation signals were bone specific. In TBL-only scaffolds, TRAP-positive staining was not associated with active mineralization labelling, indicating isolated osteoclast-mediated scaffold degradation without coupled bone formation (Fig. 4F). By contrast, the Rux-Delay group displayed co-localized ALP and TRAP staining, indicative of coupled osteoblast–osteoclast remodeling activity consistent with physiological bone repair. Quantification confirmed significantly greater osteogenic indices in Rux-Delay relative to both control conditions (Fig. 4G–J).

## Discussion

The accumulation of senescent cells and the chronic inflammatory milieu they engender through SASP secretion^33,34^ are now recognized as fundamental drivers of age-related bone healing decline, promoting osteoclast-biased remodeling and suppressing osteoblast activity^16–18^. A new framework for medical management of osteoporosis is being investigated based on senotherapeutics^35^. The present study extends this framework to the context of acute bone repair, demonstrating that the senomorphic agent Rux can accelerate healing in an aged host—but only when delivery is temporally decoupled from the acute post-injury inflammatory response. These findings carry direct implications for the clinical translation of senotherapeutic strategies to bone injury care.

**Figure 5:**
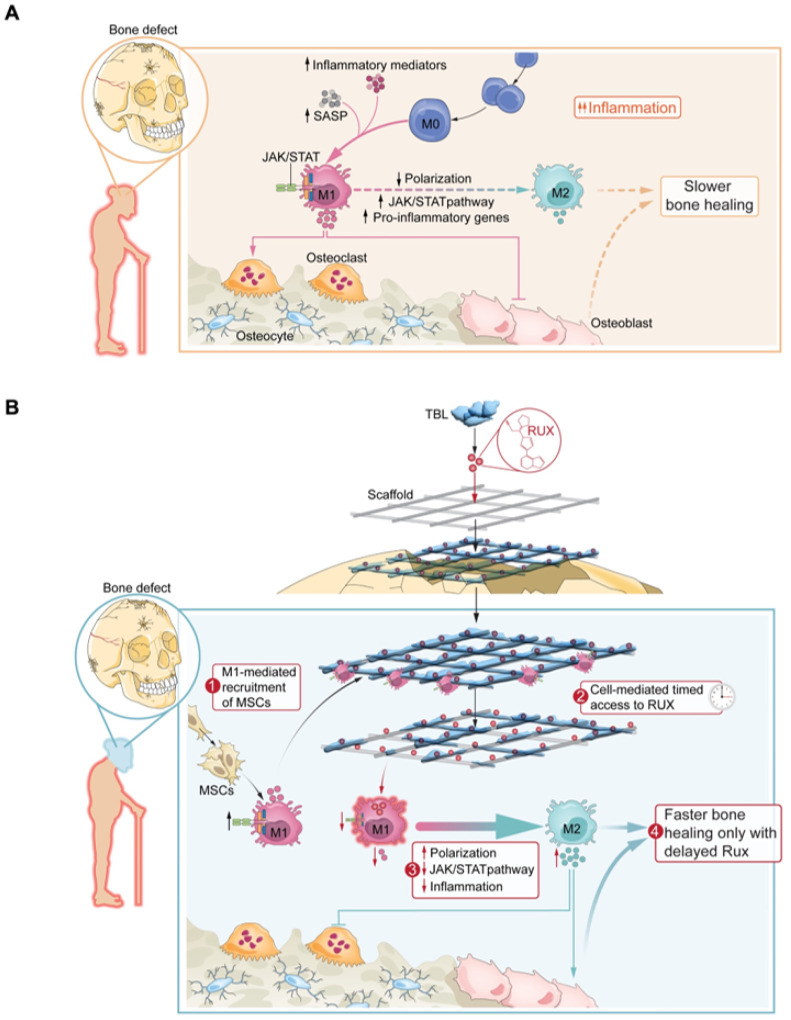
**A** Schematic illustration showing that aging impairs bone healing through upregulation of the JAK/SAT pathway. **B** Schematic illustration of TBL-mediated delivery of Rux from bioengineered bone grafts which prompts aged calvarial bone healing by modulating macrophage phenotype transitions from the M1 to M2 phenotype. Delayed delivery by the TBL prevents a premature down-regulation of the M1- associated activities of progenitor recruitment that occurs with immediate Rux. Delayed delivery by the TBL, but not immediate, enhances aged bone repair. Original artwork by Caterina Di Pietro.

The dichotomous effects of Rux observed here—detrimental when delivered immediately, beneficial when delayed—parallel the well-established timing dependence of NSAIDs and glucocorticoids on fracture healing^24,25^ and reinforce the concept that the early pro-inflammatory phase is an indispensable component of the repair program. Our gene profiling data provide mechanistic resolution of this dichotomy: immediate Rux exposure abrogates the M1-associated gene expression program within 24 hours, whereas TBL-mediated delayed release allows pro-inflammatory programing to proceed before driving a sustained shift toward reparative macrophage phenotypes at day 6. The macrophage modulating strategy proposed here is summarized in the schematic of Fig. 5. The elevated JAK/STAT pathway in aged macrophages is part of the overall aging dysfunction of aged macrophages and cytokines from the SASP of senescent cells contributes to reduced polarization to reparative phenotypes and impairs bone healing (Fig. 5A). Upon implantation, the inflammatory macrophages recruited to the TBL coated bone graft scaffold would not get access to the Rux and therefore can accomplish their role of recruiting endogenous osteoprogenitors and moderating osteoclastogenesis (Fig. 5B (1)). The timely cell-mediated access to the Rux below TBL polarizes the inflammatory macrophages to a pro-reparative phenotype by inhibition of the JAK1/JAK2 pathways upregulated in old BMDMs (Fig. 5B (2&3) resulting in faster bone repair (Fig. 5B (4)).

The biomaterial platform underpinning this approach offers several practical advantages for translational implementation. The TBL coating is formed by straightforward sequential immersion in concentrated simulated body fluid solutions, is compatible with three-dimensional printed scaffolds and other bone grafting materials and confers stability to the encased drug without requiring cold-chain handling—consistent with the remarkable durability of archeological bones and teeth as a biomolecular preservation matrix^36^. Critically, TBL delivery is particularly suited to water-insoluble drugs such as Rux, for which systemic formulation presents ongoing challenges. Drugs incorporated within calcium phosphate have been shown to have superior more effective, continuous sustained release profiles than drugs adsorbed on the exterior surface^37^. There was no observed negative effect of the calcium phosphate interaction with Rux as evidenced by the matching efficacy against the inflammatory cytokines and chemokines *Il6, Tnfa, Ccl2, Cxcl10, Cxcl11* (Fig. 2) previously shown to be downregulated by Rux administration in solution form^38^. The drug delivering bone graft can be placed by the surgeon in a clinical setting without intraoperative drug mixing, and the controlled-release kinetics eliminate the need for patients to self-administer immunomodulatory agents at precisely timed intervals following surgery—a compliance burden of particular concern in elderly populations. We envisage that surgeons could use TBL-coated grafts as a drop-in replacement for standard bone graft materials in craniofacial and orthopaedic defect repair in elderly patients, with no modification to existing surgical workflows required.

Our study highlights the value of targeting aged macrophage dysfunction as a therapeutic target for treatment of bone injuries. Most of the bone biology research examining senotherapeutic effects on bone has focused on senescent osteoblasts, osteocytes, and osteoclasts as primary cellular targets^19,39–41^. Macrophages are comparatively understudied despite being among the first cell types to respond to biomaterial implantation and playing an indispensable instructive role in the subsequent repair cascade^42,43^. Although macrophages may not themselves become senescent with age, their aging dysfunction manifests as excessive inflammatory output and impaired reparative plasticity—a phenotype that renders them logical targets for senotherapeutic intervention. Future studies should delineate more broadly the pathway components responsible for the dysregulated polarization dynamics observed in aged BMDMs and should examine whether other senomorphic or senolytic agents display comparable timing-dependent effects in the bone repair context.

Several limitations of the present study merit consideration. First, in vivo quantification of macrophage polarization kinetics by immunohistochemistry was precluded by fluorescence artifacts arising from the calcium phosphate microspheres constituting the scaffold matrix, which approximate the size of macrophages and confound cell-level imaging. Bulk RNA-sequencing of macrophages retrieved from scaffolds at early post-implantation time points—when macrophages predominate the cellular milieu—will be pursued in future work to directly validate the polarization dynamics inferred from the in vitro model. Second, the study was conducted exclusively in female mice; whether equivalent benefits of delayed Rux delivery are observed in aged male animals, which display distinct hormonal and immunological aging trajectories, remains to be established. Third, the current model does not disentangle the relative contributions of macrophage-intrinsic JAK/STAT inhibition versus suppression of paracrine SASP signals from neighboring senescent stromal cells to the observed improvements in bone repair.

In summary, the present study demonstrates that local, precision-timed delivery of a senomorphic drug from a biomimetic bone graft coating can overcome a fundamental barrier to aged bone repair: the failure of dysregulated macrophages to transit appropriately from a pro-inflammatory to a reparative phenotype. The finding that delivery timing is as important as the drug itself has broad implications, suggesting that the emerging clinical use of systemic senomorphics in osteoporosis management should be accompanied by careful attention to the timing of administration relative to any acute bone injury. More broadly, these results establish a proof-of-concept for temporally engineered immunomodulation as a strategy to rejuvenate the impaired regenerative capacity of aged tissues.

## Methods

### Transitory barrier layer (TBL) coating on disks and scaffold

The calcium phosphate-based coating process that enables delayed delivery timing is a two-step immersion process into two different 5X concentrated simulated body fluid (SBF) solutions as described previously^26,44^. The first solution, solution A, contains inhibitors for calcium phosphate crystallization, thereby forcing an amorphous calcium phosphate to be deposited which serves as a base layer supporting the growth of the crystalline bone-like calcium phosphate layer formed during immersion in solution B^29^. For the in vitro RUX-delay group, 10 ug ruxolitinib (Cat#S1378, Selleckchem, Houston, TX) in 10 ul ethanol was pipetted evenly over the surface of ultraviolet light sterilized, alumina sandblasted disks (22 mm, NUNC, Rochester, NY, USA) and allowed to dry for 10 min prior to immersion in the TBL coating solutions. To achieve Rux-immediate dosing, the TBL coating was applied to disks without Rux, cells were seeded directly on the TBL coated disks and after 4 hrs as an equivalent solution dose (10 ug/ml) was added to the cultures. For the in vivo study, the base scaffold is a three layer, 0.6 mm thick 3D printed lattice structure made of hydroxyapatite microspheres and poly-lactide-co-glycolide^45^ (Hyperelastic Bone^™^, from Dimension Bio, Chicago, USA) printed using a 250 μm nozzle in a rectilinear pattern with a 120° shift between each of the three layers. The TBL coating was applied to 3.5 mm diameter circular disks punched out of a Hyperelastic Bone^™^ sheet with a biopsy punch and UV-sterilized for 10 min on each side before TBL coating. For the Rux-delay group, Rux concentration of 40 ug in 4 ul ethanol, was pipetted evenly throughout the scaffold and allowed to dry for 10 min prior to initiating the coating process. The scaffolds with Rux applied below the TBL coating were denoted as Rux-delay. For the Rux-immediate group, the same amount of Rux (40 ug in 4 ul ethanol) was pipetted on a TBL coated scaffold immediately after it was placed into the murine calvarial bone defect.

### Mouse bone marrow derived macrophage (BMDM) cell culture

Bone marrow-derived macrophages (BMDM) were isolated from the bone marrow in femurs of female 24 month old C57BL/6 wild-type (WT) mice obtained from the National Institute on Aging mouse colony following previously published protocols^28,46^. To obtain unpolarized M0 macrophages, the cells were cultured for 7 days on 100 mm non-treated tissue culture plastic dishes in complete media (α-MEM containing 10% heated-inactivated FBS, 1% pen strip) with 20 ng/ml macrophage colony-stimulating factor (M-CSF, Invitrogen, Inc, USA, Cat# RP8615). The media was changed every 2–3 days. BMDMs were then gently dissociated by TryplE and re-suspended in complete media with 20 ng/ml M-CSF and plated at 0.5 × 10^6^ cell/ml on TBL coated disks with and without Rux in 12 well plates (N = 6). The experimental timeline is shown in Fig. 2B. A proinflammatory phenotype was induced by 24 hour stimulation with 50 ng/ml LPS and 100 ng/ml IFN-γ. Control unstimulated M0 BMDMs were also cultured for an additional 24 h. Then, for the gene expression studies, all groups had a media change and were cultured in complete media without LPS/IFNy for the remainder of the study. For the SEM morphology studies, the cells were cultured at 105,000 cells/disk and incubated with the M1 stimuli for 72 hours.

### Scanning electron microscopy

Scanning electron microscopy (SEM, Zeiss Sigma 360 VP at 3.0 kV, stage at 15 mm) was used to visualize the TBL coating morphology and the attached aged murine BMDMs. After rinsing twice with PBS, 1 ml of 2.5% glutaraldehyde in 0.1 M cacodylate buffer (No. 11653, Electron Microscopy Sciences, Hatfield, PA, USA) was added for 30 min at room temperature to fix cells, followed by rinsing three times for 5 min each in 0.1M cacodylate buffer. Then 1 ml of 1% osmium tetroxide and 0.8% Ferricyanide in 0.1M cacodylate buffer was added for thirty minutes at RT, followed by rinsing three times for 5 min each in 0.1M cacodylate buffer and three rinses in demineralized water. The disks were gradually dehydrated through a graded series of ethanol (50, 70, 95 and 3×100%) for 10 min each and critical point dried in a Leica EM CPD030 critical point dryer (Leica Microsystems, Inc) and then sputter coated with gold (Desk V, Denton Vacuum, Moorestown, NJ) for 90 s at 2mA.

### Gene expression and analysis by NanoString

RNA was extracted from cells using GeneJET RNA Purification Kit (Thermo scientific, Cat # K0732). RNA quality and concentration were measured on a Gen5 plate reader. Multiplex gene expression analysis was conducted using a custom Nanostring CodeSet with 259 genes associated with macrophage phenotype, immune response, metabolism, proliferation, extracellular matrix, and cell recruitment^30^. The gene set includes most of the genes known to be increased in aged macrophages^15^. Hybridization reactions were prepared with 50 ng of RNA for all samples. Hybridized samples were processed in the Nanostring Prep Station and Reader. Raw count data were extracted using nSolver Analysis Software.

Data normalization: Within each sample, the maximum count within the negative controls was subtracted from the raw gene counts. To normalize to positive controls, a scaling factor for each sample was calculated by dividing the geometric mean of the sample’s positive controls by the average of the geometric means. Each gene count within a sample was then divided by that sample’s scaling factor. To conduct total count normalization, the scaling factor for each sample was calculated by dividing the sum of all endogenous gene counts for that sample by the average of all sums. Each gene count within a sample was then divided by that sample’s scaling factor. Finally, all counts less than zero were changed to 1 to allow for log transformation.

Heat map clustering: Gene counts were log2-transformed, then counts within each gene were z-scored. Differentially expressed genes (DEGs) were ranked using the Differential Expression Analysis workflow in Nanostring Advanced Analysis, where p-values were adjusted using the Benjamini-Yekutieli method to control for false discovery less than 5% (q < 0.05). The heatmap function in RStudio was used to hierarchically cluster the top 30 ranked DEGs, creating dendrograms for both samples and genes. Principal components analysis: Gene counts were normalized in R Studio using the “scale” function, then a correlation matrix was generated using the “cor” function. PCA was conducted using the “princomp” function and visualized with “fviz_pca”.

### Mouse calvarial bone defect model

C57Bl/6 mice that were 21 months of age were anaesthetized with 2.5% isoflurane and a single 3.5 mm diameter critical-sized calvarial bone defect was created using a trephine bur with irrigation in the center of the right parietal bone, while avoiding the cranial suture which can inhibit bone formation. Approval for these experiments was obtained from the University of Connecticut Health Center Institutional Animal Care and Use Committee (IACUC), protocol AP-201534, prior to initiating the studies. This study is reported in accordance with ARRIVE guidelines (https://arriveguidelines.org). Defects of this size do not fully heal on their own. A 3.5 mm diameter Hyperelastic Bone^™^ with or without Rux was placed in the calvarial defect and then the periosteum and soft tissues were closed by interrupted sutures (5 − 0 Vicryl sutures, Ethicon, Somerville, NJ). Mice were given one dose of 0.08 mg/kg extended-release buprenorphine Ethiqa (Midwest Veterinary Supply, Lakeville, MN) via subcutaneous injection for pain management. Mice were housed in ventilated cages within an accredited facility under a 12-hour light/dark cycle with constant temperature (23°C) and had access to water and food ad libitum. There were six mice in each of the three groups (n = 6) (No Rux, Rux-delay and Rux Imm with randomization into groups). All mice received peritoneal cavity injections of 3 μg/g calcein and alizarin complexone calcium labels on 7 day and 1 day, respectively, prior to euthanasia to label active mineralization. At 9 weeks mice were euthanized with a Euthanex carbon dioxide induction system and calvarial bones were harvested for analysis. Upon tissue harvest, those mice in which the scaffold had moved out of the defect were excluded from further analysis. Occasionally one of the old mice died mid-study due to undiagnosed age-related issues that often occur during the aging process. The final number of mice per group at 9 weeks after surgery was n = 4.

### Bone analysis by micro-computed tomography

Mouse calvarial bones were fixed in 10% neutral buffered formalin solution for 24 hr at 4 °C prior to analysis. The volume of new mineralized bone within the calvarial bone defect sites was quantified using with a μCT40 (Scanco Medical AG, Bassersdorf, Switzerland) as described previously^47^. Serial tomographic images were acquired at 55 kV and 145 μA, collecting 1,000 projections per rotation at 300 ms integration time with 8 μm voxel size on fixed tissue samples prior to histology. Three-dimensional 16-bit grayscale images were reconstructed using standard convolution back-projection algorithms with Shepp and Logan filtering and rendered within a 16.4 mm field of view at a discrete density of 244,141 voxels/mm3 (isometric 16-μm voxels). Segmentation of bone from scaffold and marrow and soft tissue was performed in conjunction with a constrained Gaussian filter to reduce noise. New bone was characterized manually in every other 2D section as the initial defect size minus the remaining open area and then summed over the full defect depth.

### Histology and immunohistochemistry

Calvarial bones were fixed in 4% paraformaldehyde for 2 days at 4°C, rinsed with PBS and then incubated in 30% sucrose overnight. Calvarial bones were prepared for cryo-histological analysis as described previously^47^. After a section was imaged for native fluorescent signals (scaffold and bone mineral stains), the cover slide was removed and then the same section was processed for additional staining. The sections were stained for tartrate-resistant acid phosphatase staining (TRAP) activity using ELF97 (Life Tech, E6589) as the fluorescent substrate (yellow) and imaged. The same sections were next stained for alkaline phosphatase (ALP) activity using fast red substrate (Sigma, #F8764–5G) and DAPI (Mol Probes #D-1306) and re-imaged. Toluidine blue (TB) was the last stain applied (Toluidine Blue, Sigma, #T3260). Imaging of the sections was performed with the AxioImager Z1 microscope (Carl Zeiss, Thornwood, NY). Fluorescence images of the entire scaffold area were quantitatively analyzed for “% area of expression” of Calcein, Alizarin Complexone, ALP and TRAP by using the program ImageJ (www.imagej.nih.gov).

### Statistical analysis

Statistical analysis was performed using one-way ANOVA with a Tukey post-test in GraphPad Prism to conduct multiple comparisons and significance set at p < 0.05. Data were plotted as mean ± SE. For the nanostring single gene differential expression analysis was performed using multiple t-tests with FDR-adjusted p-values in GraphPad Prism. Data is represented as ± SD.

## Supplementary Files

This is a list of supplementary files associated with this preprint. Click to download.

- Supplementary.pdf

## Data Availability

The processed data for the heat map that summarizes the nanostring gene profiling is included in the supplementary material. The raw nanostring data and other data underlying this article will be shared on reasonable request to the corresponding author.
